# Treatment options for patients with large cell neuroendocrine carcinoma of the lung

**DOI:** 10.1007/s11748-014-0379-9

**Published:** 2014-04-10

**Authors:** Akira Iyoda, Takashi Makino, Satoshi Koezuka, Hajime Otsuka, Yoshinobu Hata

**Affiliations:** Division of Chest Surgery, Department of Surgery, School of Medicine, Toho University, 6-11-1 Omori-Nishi, Ota-ku, Tokyo, 143-8541 Japan

**Keywords:** Large cell neuroendocrine carcinoma, Lung, Treatment

## Abstract

Large cell neuroendocrine carcinoma (LCNEC) of the lung is categorized as a variant of large cell carcinomas, and LCNEC tumors display biological behaviors resembling those of small cell lung carcinomas and features of high-grade neuroendocrine tumors. Because patients with LCNEC have a poor prognosis, surgery alone is not sufficient. Multimodality therapies, including adjuvant chemotherapy, appear promising for improved prognosis in patients with LCNEC. In this review article, we discuss treatment options for patients with LCNEC of the lung.

## Introduction

In 1980, pulmonary neuroendocrine tumors were categorized as typical carcinoids, atypical carcinoids, or small cell lung carcinoma (SCLC) [1]. Travis et al. [2] proposed that large cell neuroendocrine carcinoma (LCNEC) should constitute a new category and reported that the prognosis of LCNEC falls between those of atypical carcinoids and SCLC. The World Health Organization [3] categorized LCNEC as non-SCLC (NSCLC). Currently, LCNEC is classified as a variant of large cell carcinomas; however, the clinical and biological characteristics of LCNEC are similar to those of SCLC. Therefore, there is still no consensus on the treatment strategy for LCNEC [4]. In this review article, we focus on treatment strategies for patients with LCNEC.

## Biological features of LCNEC

Several reports [5–7] revealed that the clinical behavior, morphology, and prognosis of LCNEC were similar to those of SCLC, even though there might be several clinicopathological differences between SCLC and LCNEC in peripheral, small-sized, high-grade neuroendocrine tumors [8]. Although Varlotto et al. [9] obtained and evaluated data on LCNEC from the Surveillance, Epidemiology, and End Results Program to show that the clinical, histopathologic, and biologic features of LCNEC were more similar to those of large cell carcinoma than SCLC, a central pathological review by an expert panel was necessary to confirm the diagnosis, a requisite lacking in the report by Varlotto et al. [10, 11]. Cytomorphologically, LCNEC shows characteristic arrangements, such as palisading or rosettes with necrosis [12]. Morphometric analysis revealed significantly different features between LCNEC and classic large cell carcinomas. Iyoda et al. [13] showed that LCNEC had significantly higher expression rates of Bcl-2 and the Ki-67 labeling index than did classic large cell carcinoma. These results revealed that the cytological and biological features of LCNEC were different from those of classic large cell carcinoma. Jones et al. [14] examined the gene expression profiles of LCNEC, SCLC, adenocarcinoma, and normal lung using microarray analysis, which was unable to distinguish LCNEC from SCLC. Using the telomeric repeat amplification protocol assay, Zaffaroni et al. [15] reported that almost all LCNEC tumors showed telomerase activity comparable to that of SCLC.

On the other hand, Nitadori et al. [16] indicated that the expression of CK7, CK18, E-cadherin, and β-catenin is more characteristic of LCNEC than of SCLC, suggesting that LCNEC and SCLC are separate entities. Ullmann et al. [17] examined comparative genomic hybridization (CGH) for LCNEC and SCLC and showed that there were differences between the two in the expression from chromosomal regions 3q, 6p, 10q, 16q, and 17p. Peng et al. [18] evaluated array-based CGH for LCNEC and SCLC and reported differences in expression at 2q, 3p, 4q, and 6p, even though LCNEC and SCLC had multiple characteristic chromosomal alterations in common. Hiroshima et al. [19] examined loss of heterozygosity (LOH) in microsatellite markers and methylation of the p16 gene in LCNEC, SCLC, and classic large cell carcinoma, and they reported that the LOH patterns of LCNEC resembled those of SCLC, even though LCNEC was also similar to classic large cell carcinoma with regard to p16 gene methylation. While many clinicopathological features of LCNEC were similar to those of SCLC, some of the biological behaviors of LCNEC differed from those of SCLC.

## Surgery for patients with LCNEC

Because most LCNECs have been diagnosed postoperatively by surgical specimens, many reports on LCNEC have referred to surgical cases, of which the majority [6, 20–27] revealed that patients with LCNEC had poor prognoses with five-year survival rates of 15–57 %. Moreover, even patients with pathological stage I LCNEC have had poor prognoses, with five-year survival rates of 27–67 % [22–26]. Iyoda et al. [28] compared the prognoses of LCNEC patients with pathological stage IA with those of patients with adenocarcinomas or squamous cell carcinomas of the same stage, and they revealed that the five-year survival rate of the LCNEC patients was 54.5 versus 89.3 % for the adenocarcinoma or squamous cell carcinoma patients. In LCNEC patients with complete resection, many recurrent tumors were observed as distant metastases [29, 30]. Therefore, surgery alone is not sufficient to treat patients with LCNEC, and subsequent adjuvant therapy may be necessary [4, 31, 32]. In 2001, Iyoda et al. [33] reported that postoperative adjuvant chemotherapy was effective in patients with LCNEC. Veronesi et al. [23] reported that 12 of 15 LCNEC cases administered induction chemotherapy were responsive (a partial response in 11 patients, a complete response in one patient) and that stage I LCNEC patients with induction or adjuvant chemotherapy tended to have a better survival than those without such therapies (*p* = 0.077). In 2005, in a retrospective study by Rossi et al. [25] adjuvant chemotherapy with CDDP+VP-16 was reported to be effective for patients with LCNEC. Iyoda et al. [34] started a prospective study of adjuvant chemotherapy in patients with LCNEC in 2000, and they selected CDDP+VP-16 as the chemotherapeutic regimen because the clinicopathologic and biological features of LCNEC are very similar to those of SCLC. They reported in 2006 that patients with adjuvant chemotherapy after complete surgical resection had a good prognosis compared with the historical control group.

Sarkaria et al. [35] reported that LCNEC had a high response rate to platinum-based neoadjuvant chemotherapy, and that resected advanced-stage patients receiving combination neoadjuvant and/or adjuvant chemotherapy may have a survival advantage. They suggested that these therapies should be considered in resectable patients with LCNEC.

Recently, many studies have reported that perioperative chemotherapy may be beneficial in patients with resected LCNEC [32, 36, 37]. Tanaka et al. [38] performed a unique study and suggested that perioperative chemotherapy might benefit the survival of patients with LCNEC if the tumors are not immunoreactive to the three neuroendocrine markers evaluated.

## Which agents are better for treatment of LCNEC patients: SCLC-based versus non-SCLC-based regimens?

When using chemotherapy, we have to consider whether to use SCLC-based or non-SCLC-based regimens for LCNEC patients, since LCNEC is currently classified as non-SCLC, even though the clinical and biological characteristics of LCNEC are similar to those of SCLC.

Sun et al. [39] revealed that the response rate to platinum-based chemotherapy was 60 %, whereas the response rate to non-platinum-based chemotherapy was 11 %. Moreover, they examined whether advanced LCNEC should be treated similarly to SCLC versus non-SCLC with respect to chemotherapeutic regimens, and they concluded that advanced LCNEC could be treated appropriately in a manner similar to SCLC rather than NSCLC. Igawa et al. [40] reported that the effectiveness of chemotherapy for unresectable LCNEC was comparable to that for extended disease SCLC. Tokito et al. [41] reported that the response rate for SCLC-based chemotherapy was 70 % in patients with LCNEC. Shimada et al. [42] suggested that the overall response rate to initial chemotherapy or chemoradiotherapy and the survival outcomes of high-grade neuroendocrine carcinoma-probable LCNEC were comparable to those of SCLC, even though the efficacy of second-line chemotherapy might differ between LCNEC and SCLC. Yamazaki et al. [43] reported that the response rate of LCNEC to cisplatin-based chemotherapy was comparable with that of SCLC.

## Surgical indication for patients with LCNEC

Treut et al. [44] performed a multicenter phase II study of cisplatin–etoposide chemotherapy for stage IIIB or IV LCNEC patients, and they concluded that the outcome of advanced LCNEC patients treated with cisplatin–etoposide doublet chemotherapy was poor, comparable with that of patients with advanced SCLC, with an 8-month median overall survival. Their study revealed that the multi-institutional trials on LCNEC, especially on treatments such as chemotherapy, were necessary to have the centralized pathologist review because their study reclassified 11 (27.5 %) of 40 LCNEC patients into 9 as SCLC, 1 as undifferentiated non-SCLC, and 1 as atypical carcinoid. These difficulties may also affect the result of clinical trials using adjuvant chemotherapy for LCNEC patients. Niho et al. [45] performed combination chemotherapy with irinotecan and cisplatin in patients with advanced LCNEC and revealed that the response rate was 46.7 % in the LCNEC group. They concluded that combination chemotherapy with irinotecan and cisplatin was an active regimen in patients with LCNEC, although the response rate and the overall survival period in LCNEC patients were inferior to those of SCLC patients. Patients with advanced-stage LCNEC had a poor prognosis because they could not always achieve a complete response. Although there were high response rates for platinum-based and SCLC-based chemotherapies, almost all patients had only partial responses. Patients with LCNEC may not be able to expect complete responses with platinum-based and SCLC-based chemotherapies compared with SCLC patients, even though these chemotherapies are as effective as adjuvant treatment after removing tumors.

The indication for surgery is limited to stage I in patients with SCLC, however, surgery with adjuvant chemotherapy may achieve satisfactory results in terms of survival in regard to surgical indications for patients with not only stage I but also stage II/III in LCNEC [46]. Iyoda et al. [29] reported that platinum-based adjuvant chemotherapy after surgery might be useful for preventing recurrence in patients with LCNEC. Therefore, surgical indications for patients with LCNEC may not be limited to clinical stage I cases, and surgery with adjuvant chemotherapy should be attempted for resectable LCNEC.

## New treatments for LCNEC

Many reports revealed that platinum-based and SCLC-based chemotherapies were effective for patients with LCNEC. However, those results were not enough to improve the prognoses of patients with LCNEC, especially those with advanced stages. Therefore, we need to discover new treatments for patients with LCNEC.

As for new chemotherapeutic agents, Kenmotsu et al. [47] reported that nedaplatin plus irinotecan was effective and safe for patients with LCNEC. Yoshida et al. [48] studied the effectiveness of amrubicin monotherapy in patients with previously treated advanced LCNEC, for whom they showed that amrubicin was potentially active, yielding an objective response rate of 27.7 %.

With molecular targeted therapies, De Pas et al. [49] reported a case of LCNEC with EGFR mutation, which had a dramatic response to gefitinib. Kozuki et al. [32] also reported a case with a partial response to gefitinib. Other authors [50–52] reported cases of LCNEC with EGFR mutation, but only a few [51, 53]. Even though few patients have EGFR mutations, EGFR-TKI may be an optimal treatment for certain patients with LCNEC. Filosso et al. [54] concluded that octreotide, which is used for the treatment of carcinoid syndrome, was effective for patients with LCNEC.

In experimental studies, Odate et al. [55] found that the expression of tropomyosin-related kinase B and brain-derived neurotrophic factor was significantly higher in LCNEC than in SCLC, and they proposed that these two genes might be potential targets in LCNEC. Iyoda et al. [53] examined new markers for patients with LCNEC, and they suggested a potential role for anti-VEGF, anti-c-KIT, and possibly anti-HER2-targeted agents in the treatment of LCNEC. Yokouchi et al. [56] reported successful treatment with intravitreal injection of bevacizumab in LCNEC patients with metastasis to the iris.

## Treatments for patients with LCNEC originating from other organs

The first report of LCNEC was in the lung, and most reports have also referred to pulmonary LCNECs. However, we may be able to get important information regarding treatment options for lung LCNEC from studies on treatments for patients with LCNEC of other organs.

Kusafuka et al. [57] reported a trend toward recommending primary chemotherapy and radiation, rather than primary surgery with postoperative radiation/systemic therapy, for patients with LCNEC in the head and neck, based on the fact that LCNEC has extremely similar clinical features, patterns and timing of spread, and prognosis as those of small cell carcinomas, suggesting that treatment should also be similar. Ose et al. [58] reported a case of thymic LCNEC administered induction chemoradiation therapy (three cycles of cisplatin/etoposide and 45 Gy of hyperfractionated radiation) because of invasion to the aortic arch and pulmonary trunk. Pathologic examination revealed no viable cells in the resected tumor, and the patient was alive 3 years later without recurrence. Tangjitgamol et al. [59] reported a case of LCNEC of the uterine cervix with good response to primary chemotherapy of paclitaxel/carboplatin. Embry et al. [60] suggested that LCNEC of the cervix was more aggressive with a poorer prognosis than squamous cell carcinoma, and perioperative chemotherapy, platinum with or without etoposide, improved survival of patients with LCNEC of the cervix. Yoseph et al. [61] conducted a review of more than 70 cases with LCNEC of the uterine cervix and found that current multimodal strategies have been adapted mainly from treatments used for neuroendocrine carcinomas of the lung, even though no consensus had been reached as far as an optimal treatment plan because of the rarity of cervical LCNEC. Oberstein et al. [62] reported that gastric LCNEC is a high-grade tumor with an aggressive course, and that treatment with platinum-based chemotherapy might provide clinical benefits. Evans et al. [63] reported seven cases with prostate LCNEC, of which all six cases with complete follow-up died from widespread metastases, and five of these six cases were treated with platinum-based chemotherapy, with a mean survival of 7 months from the time of the last course. Shimono et al. [64] reported that multimodal treatment, including surgery, chemotherapy with cisplatin and etoposide, and radiation therapy, achieved a good response and long survival in a patient with LCNEC of the gallbladder.

Although we are beginning to understand the clinicopathological and biological features of patients with LCNEC little by little, there is no consensus on treatment in patients with lung LCNEC. We need large-scale trials in order to reach a consensus; however, in the near future, we may not be able to perform such trials because of the rarity of LCNEC cases. Therefore, we should continue trying to obtain useful information from several small-scale studies as well as to evaluate new strategies, including molecular targeted therapies, for patients with LCNEC.

## References

[1] Arrigoni MG, Woolner LB, Bernatz PE (1972). Atypical carcinoid tumors of the lung. J Thorac Cardiovasc Surg.

[2] Travis WD, Linnoila RI, Tsokos MG, Hitchcock CL, Cutler GB, Nieman L (1991). Neuroendocrine tumors of the lung with proposed criteria for large-cell neuroendocrine carcinoma. Am J Surg Pathol.

[3] World Health Organization (1999). Histological typing of lung and pleural tumours.

[4] Iyoda A, Hiroshima K, Nakatani Y, Fujisawa T (2007). Pulmonary large cell neuroendocrine carcinoma—its place in the spectrum of pulmonary carcinoma. Ann Thorac Surg.

[5] Travis WD, Rusch W, Flieder DB, Falk R, Fleming MV, Gal AA (1998). Survival analysis of 200 pulmonary neuroendocrine tumors with clarification of criteria for atypical carcinoid and its separation from typical carcinoid. Am J Surg Pathol.

[6] Asamura H, Kameya T, Matsuno Y, Noguchi M, Tada H, Ishikawa Y (2006). Neuroendocrine neoplasms of the lung: a prognostic spectrum. J Clin Oncol.

[7] Iyoda A, Hiroshima K, Baba M, Saitoh Y, Ohwada H, Fujisawa T (2002). Pulmonary large cell carcinomas with neuroendocrine features are high grade neuroendocrine tumors. Ann Thorac Surg.

[8] Isaka M, Nakagawa K, Ohde Y, Okumura T, Watanabe R, Ito I (2012). A clinicopathological study of peripheral, small-sized high-grade neuroendocrine tumours of the lung: differences between small-cell lung carcinoma and large-cell neuroendocrine carcinoma. Eur J Cardiothorac Surg.

[9] Varlotto JM, Medford-Davis LN, Recht A, Flickinger JC, Schaefer E, Zander DS (2011). Should large cell neuroendocrine lung carcinoma be classified and treated as a small cell lung cancer or with other large cell carcinomas?. J Thorac Oncol.

[10] Asamura H (2011). Identity, similarity, and difference between large cell neuroendocrine carcinoma and small cell carcinoma. J Thorac Oncol.

[11] Rossi G, Mengoli MC, Cavazza A (2011). Pulmonary large cell neuroendocrine carcinoma: a true high-grade neuroendocrine tumor needing prospective therapeutic data. J Thorac Oncol.

[12] Iyoda A, Baba M, Hiroshima K, Saitoh H, Moriya Y, Shibuya K (2004). Imprint cytologic features of pulmonary large cell neuroendocrine carcinoma: comparison with classic large cell carcinoma. Oncol Rep.

[13] Iyoda A, Hiroshima K, Moriya Y, Mizobuchi T, Otsuji M, Sekine Y (2004). Pulmonary large cell neuroendocrine carcinoma demonstrates high proliferative activity. Ann Thorac Surg.

[14] Jones MH, Virtanen C, Honjoh D, Miyoshi T, Satoh Y, Okumura S (2004). Two prognostically significant subtypes of high-grade lung neuroendocrine tumours independent of small-cell and large-cell neuroendocrine carcinomas identified by gene expression profiles. Lancet.

[15] Zaffaroni N, De Polo D, Villa R, Della Porta C, Collini P, Fabbri A (2003). Differential expression of telomerase activity in neuroendocrine lung tumours: correlation with gene product immunophenotyping. J Pathol.

[16] Nitadori J, Ishii G, Tsuta K, Yokose T, Murata Y, Kodama T (2006). Immunohistochemical differential diagnosis between large cell neuroendocrine carcinoma and small cell carcinoma by tissue microarray analysis with a large antibody panel. Am J Clin Pathol.

[17] Ullmann R, Petzmann S, Sharma A, Cagle PT, Popper HH (2001). Chromosomal aberrations in a series of large-cell neuroendocrine carcinomas: unexpected divergence from small-cell carcinoma of the lung. Hum Pathol.

[18] Peng WX, Shibata T, Katoh H, Kokubu A, Matsuno Y, Asamura H (2005). Array-based comparative genomic hybridization analysis of high-grade neuroendocrine tumors of the lung. Cancer Sci.

[19] Hiroshima K, Iyoda A, Shibuya K, Haga Y, Toyozaki T, Iizasa T (2004). Genetic alterations in early-stage pulmonary large cell neuroendocrine carcinoma. Cancer.

[20] Iyoda A, Hiroshima K, Toyozaki T, Haga Y, Fujisawa T, Ohwada H (2001). Clinical characterization of pulmonary large cell neuroendocrine carcinoma and large cell carcinoma with neuroendocrine morphology. Cancer.

[21] Jiang SX, Kameya T, Shoji M, Dobashi Y, Shinada J, Yoshimura H (1998). Large cell neuroendocrine carcinoma of the lung. A histologic and immunohistochemical study of 22 cases. Am J Surg Pathol.

[22] Takei H, Asamura H, Maeshima A, Suzuki K, Kondo H, Niki T (2002). Large cell neuroendocrine carcinoma of the lung: a clinicopathologic study of eighty–seven cases. J Thorac Cardiovasc Surg.

[23] Veronesi G, Morandi U, Alloisio M, Terzi A, Cardillo G, Filosso P (2006). Large cell neuroendocrine carcinoma of the lung: a retrospective analysis of 144 surgical cases. Lung Cancer.

[24] Paci M, Cavazza A, Annessi V, Putrino I, Ferrari G, De Franco S (2004). Large cell neuroendocrine carcinoma of the lung: a 10-year clinicopathologic retrospective study. Ann Thorac Surg.

[25] Rossi G, Cavazza A, Marchioni A, Longo L, Migaldi M, Sartori G (2005). Role of chemotherapy and the receptor tyrosine kinases KIT, PDGFRalpha, PDGFRbeta, and Met in large-cell neuroendocrine carcinoma of the lung. J Clin Oncol.

[26] Battafarano RJ, Fernandez FG, Ritter J, Meyers BF, Guthrie TJ, Cooper JD (2005). Large cell neuroendocrine carcinoma: an aggressive form of non-small cell lung cancer. J Thorac Cardiovasc Surg.

[27] Skuladottir H, Hirsch FR, Hansen HH, Olsen JH (2002). Pulmonary neuroendocrine tumors: incidence and prognosis of histological subtype: a population-based study in Denmark. Lung Cancer.

[28] Iyoda A, Hiroshima K, Moriya Y, Sekine Y, Shibuya K, Iizasa T (2006). Prognostic impact of large cell neuroendocrine histology in patients with pathological stage 1a pulmonary non-small cell carcinoma. J Thorac Cardiovasc Surg.

[29] Iyoda A, Hiroshima K, Moriya Y, Iwadate Y, Takiguchi Y, Uno T (2009). Postoperative recurrence and the role of adjuvant chemotherapy in patients with pulmonary large-cell neuroendocrine carcinoma. J Thorac Cardiovasc Surg.

[30] Iyoda A, Jiang SX, Travis WD, Kurouzu N, Ogawa F, Amano H (2013). Clinicopathological features and the impact of the new TNM classification of malignant tumors in patients with pulmonary large cell neuroendocrine carcinoma. Mol Clin Oncol.

[31] Fernandez FG, Battafarano RJ (2006). Large-cell neuroendocrine carcinoma of the lung. Cancer Control.

[32] Kozuki T, Fujimoto N, Ueoka H, Kiura K, Fujiwara K, Shiomi K (2005). Complexity in the treatment of pulmonary large cell neuroendocrine carcinoma. J Cancer Res Clin Oncol.

[33] Iyoda A, Hiroshima K, Toyozaki T, Haga Y, Baba M, Fujisawa T (2001). Adjuvant chemotherapy for large cell carcinoma with neuroendocrine features. Cancer.

[34] Iyoda A, Hiroshima K, Moriya Y, Takiguchi Y, Sekine Y, Shibuya K (2006). Prospective study of adjuvant chemotherapy for pulmonary large cell neuroendocrine carcinoma. Ann Thorac Surg.

[35] Sarkaria IS, Iyoda A, Roh MS, Sica G, Kuk D, Sima CS (2011). Neoadjuvant and adjuvant chemotherapy in resected pulmonary large cell neuroendocrine carcinomas: a single institution experience. Ann Thorac Surg.

[36] Saji H, Tsuboi M, Matsubayashi J, Miyajima K, Shimada Y, Imai K (2010). Clinical response of large cell neuroendocrine carcinoma of the lung to perioperative adjuvant chemotherapy. Anticancer Drugs.

[37] Abedallaa N, Tremblay L, Baey C, Fabre D, Planchard D, Pignon JP (2012). Effect of chemotherapy in patients with resected small-cell or large-cell neuroendocrine carcinoma. J Thorac Oncol.

[38] Tanaka Y, Ogawa H, Uchino K, Ohbayashi C, Maniwa Y, Nishio W (2013). Immunohistochemical studies of pulmonary large cell neuroendocrine carcinoma: a possible association between staining patterns with neuroendocrine markers and tumor response to chemotherapy. J Thorac Cardiovasc Surg.

[39] Sun JM, Ahn MJ, Ahn JS, Um SW, Kim H, Kim HK (2012). Chemotherapy for pulmonary large cell neuroendocrine carcinoma: similar to that for small cell lung cancer or non-small cell lung cancer?. Lung Cancer.

[40] Igawa S, Watanabe R, Ito I, Murakami H, Takahashi T, Nakamura Y (2010). Comparison of chemotherapy for unresectable pulmonary high-grade non-small cell neuroendocrine carcinoma and small-cell lung cancer. Lung Cancer.

[41] Tokito T, Kenmotsu H, Watanabe R, Ito I, Shukuya T, Ono A, et al. Comparison of chemotherapeutic efficacy between LCNEC diagnosed using large specimens and possible LCNEC diagnosed using small biopsy specimens. Int J Clin Oncol. 2012;19 (Epub ahead of print). 10.1007/s10147-012-0509-2 23250620

[42] Shimada Y, Niho S, Ishii G, Hishida T, Yoshida J, Nishimura M (2012). Clinical features of unresectable high-grade lung neuroendocrine carcinoma diagnosed using biopsy specimens. Lung Cancer.

[43] Yamazaki S, Sekine I, Matsuno Y, Takei H, Yamamoto N, Kunitoh H (2005). Clinical responses of large cell neuroendocrine carcinoma of the lung to cisplatin-based chemotherapy. Lung Cancer.

[44] Le Treut J, Sault MC, Lena H, Souquet PJ, Vergnenegre A, Le Caer H (2013). Multicentre phase II study of cisplatin-etoposide chemotherapy for advanced large-cell neuroendocrine lung carcinoma: the GFPC 0302 study. Ann Oncol.

[45] Niho S, Kenmotsu H, Sekine I, Ishii G, Ishikawa Y, Noguchi M (2013). Combination chemotherapy with irinotecan and cisplatin for large-cell neuroendocrine carcinoma of the lung: a multicenter phase II study. J Thorac Oncol.

[46] Fournel L, Falcoz PE, Alifano M, Charpentier MC, Boudaya MS, Magdeleinat P (2013). Surgical management of pulmonary large cell neuroendocrine carcinomas: a 10-year experience. Eur J Cardiothorac Surg.

[47] Kenmotsu Y, Oshita F, Sugiura M, Murakami S, Kondo T, Saito H (2012). Nedaplatin and irinotecan in patients with large-cell neuroendocrine carcinoma of the lung. Anticancer Res.

[48] Yoshida H, Sekine I, Tsuta K, Horinouchi H, Nokihara H, Yamamoto N (2011). Amrubicin monotherapy for patients with previously treated advanced large-cell neuroendocrine carcinoma of the lung. Jpn J Clin Oncol.

[49] De Pas TM, Giovannini M, Manzotti M, Trifirò G, Toffalorio F, Catania C (2011). Large-cell neuroendocrine carcinoma of the lung harboring EGFR mutation and responding to gefitinib. J Clin Oncol.

[50] Yanagisawa S, Morikawa N, Kimura Y, Nagano Y, Murakami K, Tabata T (2012). Large-cell neuroendocrine carcinoma with epidermal growth factor receptor mutation: possible transformation of lung adenocarcinoma. Respirology.

[51] Sakai Y, Yamasaki T, Kusakabe Y, Kasai D, Kotani Y, Nishimura Y (2013). Large-cell neuroendocrine carcinoma of lung with epidermal growth factor receptor (EGFR) gene mutation and co-expression of adenocarcinoma markers: a case report and review of the literature. Multidiscip Respir Med.

[52] Yoshida Y, Ota S, Murakawa T, Takai D, Nakajima J. Combined large cell neuroendocrine carcinoma and adenocarcinoma with epidermal growth factor receptor mutation in a female patient who never smoked. Ann Thorac Cardiovasc Surg. 2013 (Epub ahead of print). 10.5761/atcs.cr.12.02217 23518631

[53] Iyoda A, Travis WD, Sarkaria IS, Jiang SX, Amano H, Sato Y (2011). Expression profiling and identification of potential molecular targets for therapy in pulmonary large-cell neuroendocrine carcinoma. Exp Ther Med.

[54] Filosso PL, Ruffini E, Oliaro A, Rena O, Casadio C, Mancuso M (2005). Large-cell neuroendocrine carcinoma of the lung: a clinicopathologic study of eighteen cases and the efficacy of adjuvant treatment with octreotide. J Thorac Cardiovasc Surg.

[55] Odate S, Nakamura K, Onishi H, Kojima M, Uchiyama A, Nakano K (2013). TrkB/BDNF signaling pathway is a potential therapeutic target for pulmonary large cell neuroendocrine carcinoma. Lung Cancer.

[56] Yokouchi H, Kitahashi M, Oshitari T, Yamamoto S (2013). Intravitreal bevacizumab for iris tumor metastasized from large cell neuroendocrine carcinoma of lung. Graefes Arch Clin Exp Ophthalmol.

[57] Kusafuka K, Ferlito A, Lewis JS, Woolgar JA, Rinaldo A, Slootweg PJ (2012). Large cell neuroendocrine carcinoma of the head and neck. Oral Oncol.

[58] Ose N, Inoue M, Morii E, Shintani Y, Sawabata N, Okumura M (2013). Multimodality therapy for large cell neuroendocrine carcinoma of the thymus. Ann Thorac Surg.

[59] Tangjitgamol S, Manusirivithaya S, Choomchuay N, Leelahakorn S, Thawaramara T, Pataradool K (2007). Paclitaxel and carboplatin for large cell neuroendocrine carcinoma of the uterine cervix. J Obstet Gynaecol Res.

[60] Embry JR, Kelly MG, Post MD, Spillman MA (2011). Large cell neuroendocrine carcinoma of the cervix: prognostic factors and survival advantage with platinum chemotherapy. Gynecol Oncol.

[61] Yoseph B, Chi M, Truskinovsky AM, Dudek AZ (2012). Large-cell neuroendocrine carcinoma of the cervix. Rare Tumors.

[62] Oberstein PE, Kenney B, Krishnamoorthy SK, Woo Y, Saif MW (2012). Metastatic gastric large cell neuroendocrine carcinoma: a case report and review of literature. Clin Colorectal Cancer.

[63] Evans AJ, Humphrey PA, Belani J, van der Kwast TH, Srigley JR (2006). Large cell neuroendocrine carcinoma of prostate: a clinicopathologic summary of 7 cases of a rare manifestation of advanced prostate cancer. Am J Surg Pathol.

[64] Shimono C, Suwa K, Sato M, Shirai S, Yamada K, Nakamura Y (2009). Large cell neuroendocrine carcinoma of the gallbladder: long survival achieved by multimodal treatment. Int J Clin Oncol.

